# Trends in the Incidence of Cardiovascular Diagnoses and Procedures over the Years 2012–2021 in Israel: The Impact of the COVID-19 Pandemic

**DOI:** 10.3390/jcm13020476

**Published:** 2024-01-15

**Authors:** Orna Reges, Wiessam Abu Ahmad, Erez Battat, Walid Saliba, Yael Wolff Sagy, Asaf Danon, Gil Lavie

**Affiliations:** 1 Department of Health Systems Management, School of Health Sciences, Ariel University, Ariel 4077625, Israel; 2 Branch of Planning and Strategy, Clalit Health Services, Tel Aviv 6209804, Israel; wisamabu@clalit.org.il (W.A.A.); erezb@clalit.org.il (E.B.); yaelwo@clalit.org.il (Y.W.S.); gillav@clalit.org.il (G.L.); 3 Hadassah Braun School of Public Health and Community Medicine, Hebrew University, Jerusalem 9112102, Israel; 4 Department of Community Medicine and Epidemiology, Carmel Medical Center, Haifa 3436212, Israel; saliba_wa@clalit.org.il; 5 Ruth and Bruce Rappaport Faculty of Medicine, Technion—Israel Institute of Technology, Haifa 3200003, Israel; asafda@clalit.org.il; 6 Department of Cardiology, Carmel Medical Center, Haifa 3436212, Israel

**Keywords:** cardiovascular disease, COVID-19, trends

## Abstract

Prior studies found reduced incidences of cardiovascular diagnoses and treatments in the initial phase of the COVID-19 pandemic. However, these studies included a limited number of outcomes and did not consider pre-pandemic trends. This study aimed to describe trends in the incidence of cardiovascular diagnoses and treatments over the years 2012–2021 in Israel and to compare the two years of the COVID-19 period with the preceding 8 years. In this retrospective, population-based study, carried out within Clalit Health Services, the incidence rates of cardiovascular outcomes were calculated for individuals aged ≥ 25 (~2.7 million adults per year) during the first (Y1, 3/2020–2/2021) and second (Y2, 3/2021–2/2022) years of COVID-19 and the 8 years prior (3/2012–2/2020). Declines were observed in Y1 compared to 2019 in all diagnoses and treatments: STEMI (−16.3%; 95% CI: −16.6, −16.1), non-STEMI (−16.4%; −16.6, −16.2), AF (−14.1%; −14.2, −14.0), CHF (−7.8%; −7.9, −7.7), CVA (−5.0%; −5.0, −4.9), catheterization (−64.7%; −65.2, −64.2), CABG (−77.7%; −79.2, −76.2), ablation (−21.2%; −22.0, −20.4), pacemaker implantation (−39.3%; −40.7, −37.9), and defibrillator insertion (−12.5%; −13.1, −12.0). Compared with expected rates based on pre-pandemic trends, observed rates were within expected ranges (CHF, CVA, and ablation), less than expected (STEMI, non-STEMI, AF, catheterization, CABG, and pacemaker insertion), or more than expected (defibrillator insertion). In Y2, STEMI, catheterization, and CABG returned to expected rates; non-STEMI and AF were lower than expected; and CHF, CVA, ablation, and pacemaker and defibrillator implantations were higher than expected. Several cardiovascular diagnoses and treatment trends were interrupted by COVID-19. The long-term consequences of these changes should be considered by health policymakers.

## 1. Introduction

The worldwide outbreak of the coronavirus disease in 2019 (COVID-19) and the rapid growth of cases around the world had a huge impact on healthcare provision and utilization. The first step for most health services was accelerating the use of telemedicine and reducing unnecessary in-person visits [1,2,3]. Given the continuous lockdowns and the fear of contagion, many people avoided the use of medical services during the pandemic [4,5]. Previous studies on the cardiovascular sequelae of the COVID-19 pandemic mostly reported decreases in cardiovascular diagnoses and medical procedures in the initial phase of the pandemic [6,7,8,9,10]. Different trends were observed as the pandemic proceeded [11]. Those studies, however, mostly reported a limited number of outcomes, or compared the COVID-19 period to a short preceding period, without considering pre-pandemic trends. Assessing cardiovascular-related outcomes in the contexts of the pre-pandemic trends may provide a more accurate evaluation of the consequences of the pandemic. The objectives of this study were to describe trends in the incidence of cardiovascular diagnoses and procedures over the years 2012–2021 in Israel and to compare the first two years of the COVID-19 period with the preceding 8 years.

## 2. Materials and Methods

This study was approved by the Clalit institutional review board. Since this study was based on historical data only, participants’ consent was not required. All patient identities were concealed.

### 2.1. Study Design and Source Population

This was a retrospective, population-based study of Clalit Health Services (Clalit) members aged ≥ 25 years during the first 2 years of the COVID-19 pandemic and the preceding 8 years. To separate between the pandemic and the pre-pandemic periods in Israel, each year was defined as running from March to February of the following year, and incidence rates of cardiovascular diagnoses and procedures were calculated for each year during the decade.

In Israel, health insurance is universal and mandatory. Each Israeli is required to choose one of four health maintenance organizations (HMOs), which are obliged to accept all registrations. All four HMOs provide an identical basic basket of health services, by law. Clalit, the largest HMO in Israel, provides inpatient and outpatient services for approximately 4.8 million members, or 52% of the Israeli population. Clalit owns and operates 14 hospitals, approximately 1500 clinics nationwide, as well as laboratories, imaging institutes, and pharmacies. The ability to integrate outpatient data with inpatient data enhances the accuracy and validity of identifying diagnoses and procedures [12]. Members of Clalit may receive inpatient health services in hospitals which are not operated by Clalit. Services that are provided to Clalit members by non-Clalit public hospitals in Israel are reported to Clalit on a regular basis, including diagnoses and procedures, in order to ensure the continuity of care and the proper documentation of patients’ electronic medical records. Furthermore, Clalit maintains registries of chronic diseases that are consistently optimized for use in clinical decisions and research purposes. This, together with a low annual turnover of approximately 1–2% of Clalit members, enables the organization to maintain an extensive longitudinal electronic health services data warehouse which covers more than two decades and follows each member during this time span.

### 2.2. Variables

Data on new cardiovascular diagnoses and procedures during the first year of COVID-19 (Y1, 3/2020–2/2021), the second year of COVID-19 (Y2, 3/2021–2/2022), and each of the prior 8 years (3/2012–2/2020) were extracted from both inpatient and outpatient medical records. Cardiovascular diagnoses and procedures were determined using the International Classification of Diseases, 9th revision (ICD-9), the coding system currently employed in Israel. Incidence of the following diagnoses was included: ST-elevation myocardial infarction (STEMI), non-ST-elevation myocardial infarction (non-STEMI), atrial fibrillation (AF), congestive heart failure (CHF), and cerebrovascular accident (CVA). The following procedures were included: interventional coronary catheterization, coronary artery bypass graft (CABG), ablation, pacemaker insertion, and defibrillator insertion. The source of information and the ICD-9 codes of the medical diagnoses and procedures are presented in Supplementary Table S1.

### 2.3. Statistical Analysis

Trends in patients’ sex and age were examined in the overall population to assess whether the trends in the outcomes of interest were related to changes in Clalit’s member composition.

Monthly and annual incidence rates (per 100,000 members) of cardiovascular diagnoses and procedures were calculated for each year during 2012–2021. To quantify the uncertainty around the estimated risk, 95% confidence intervals (CIs) were determined for yearly rates. The Wilson score interval method was applied to compute the lower and upper bounds of the CIs. 

The Cochran–Armitage test for trend was utilized to assess the trends in CVD outcomes during the 8 years prior to the COVID-19 outbreak. This test is particularly suited for detecting trends in binary outcomes across ordered categories. 

The incidence of each CVD diagnosis and procedure during the pandemic period was compared to both the year prior to the outbreak of COVID-19 (2019) and the trend observed in the preceding eight years. Specifically, risk difference (RD) was computed as the absolute difference in risks between the examined year and 2019. A 95% CI for the RD was established using the delta method, which included calculating the standard error of the RD and using it to define the lower and upper bounds of the interval. 

To evaluate whether the data for 2020 and 2021 deviated from the trend observed in the years 2012–2019, a linear regression line was fitted to the annual data. Predicted risks were then calculated for the years 2020–2021 and subsequently compared to the actual data for those two years. Deviation from the expected trend was considered when the predicted risk was not included in the 95% CI of the observed rate.

Additionally, change-point analysis (CPA) was conducted to identify abrupt changes in the monthly incidence rates that occurred over the decade, with a particular focus on the initiation of the pandemic. A change point is defined as a shift in the mean incidence. A binary segmentation algorithm was used to locate multiple change points, with the maximum number of five change points set for each outcome. These analyses were performed using the ‘ggchangepoint’ package in R-Studio, version 2022.02.0, which utilizes a non-parametric test to detect change points. All analyses were conducted using R statistical software, version 3.5.0 (R Project for Statistical Computing).

## 3. Results

Over the years 2012 to 2021, there were an average of 2.7 million Clalit members aged ≥25 per year. Sex distribution and mean age were similar over the years (Table 1).

### 3.1. Pre-Pandemic Trends

**Figure 1:**
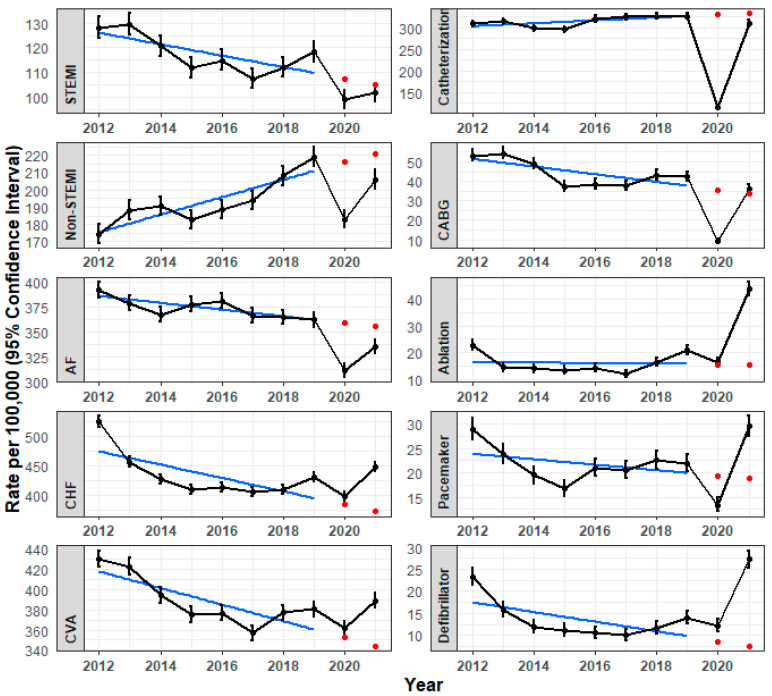
Annual incidence rates of cardiovascular procedures during the years 2012–2021 (per 100,000 patients, black line). Blue line indicates the overall trend between 2012 and 2019, while the red dots represent the expected continuation of that trend in 2020 and 2021.

During the 8 years prior to the COVID-19 outbreak (2012–2019), significant decreases in the incidence rates of some cardiovascular diagnoses were observed, notably in STEMI, CHF, and CVA. The incidence rates of AF decreased only moderately over the years, and those of non-STEMI increased (Table 2, blue line in Figure 1, *p* < 0.001 for all diagnoses). For the reported cardiovascular procedures, decreases in the annual rates of CABG and defibrillator insertion were observed over the 8 years prior to the COVID-19 outbreak (*p* < 0.001). Overall, the trends for cardiac catheterization, ablation, and pacemaker insertion were relatively stable (Table 3, blue line in Figure 1).

### 3.2. The First Year of the COVID-19 Pandemic (Y1)

When comparing the observed incidence rates of CVD diagnoses and procedures to those in the year prior to the COVID-19 outbreak in Israel (3/2019–2/2020), significant declines were demonstrated in all cardiovascular diagnoses during Y1 (Table 2). Specifically, a decline was observed in STEMI (−16.3%, 95% CI: −16.6% to −16.1%; −19.3/100,000), non-STEMI (−16.4%, 95% CI: −16.6% to −16.2%; −35.9/100,000), AF (−14.1% to 95% CI: −14.2%, −14.0%; −51.0/100,000), CHF (−7.8%, 95% CI: −7.9% to −7.7%; −33.6/100,000), and CVA (−5.0%, 95% CI: −5.0% to −4.9%; −19.0/100,000).

Significant declines were also observed in all annual rates of cardiovascular procedures during Y1 (Table 3), including a decline in catheterization (−64.7%, 95% CI: −65.2% to −64.2%; −211.7.0/100,000), CABG (−77.7%, 95% CI: −79.2% to −76.2%; 32.9/100,000), ablation (−21.2%, 95% CI: −22.0% to −20.4%; −4.4/100,000), pacemaker implantation (−39.3%, 95% CI: −40.7% to −37.9%; −8.6/100,000), and defibrillator insertion (−12.5%, 95% CI: −13.1% to −12.0%; −1.7/100,000).

CPA revealed significant change points in the beginning of the COVID-19 pandemic in Israel (February 2020), with a decrease in the incidences of STEMI, non-STEMI, AF, CVA, catheterization, and CABG.

### 3.3. The Second Year of the COVID-19 Pandemic (Y2)

In the second year of the pandemic, increases were observed in all diagnoses and procedures when compared to Y1. However, rates of these events differed in regard to the baseline (2019). Specifically, the declines observed during Y1 were preserved but attenuated in Y2 for diagnoses of STEMI (−13.9%, 95% CI: −14.1 to −13.7; −16.4), non-STEMI (−5.9%, 95% CI: −5.9% to −5.8%; −12.8), and AF (−7.5%, 95% CI: −7.6 to −7.5; −27.2). The incidence rates of CHF and CVA came very close to the baseline during Y2 (Table 2). For the procedures in Y2, a moderated but significant decrease in CABG compared to the baseline was still observed (−15.0%, 95% CI: −15.4 to −14.6; −6.3). In catheterization, incidence rate was similar to the baseline. However, sharp increases were observed in ablation (111.1%, 95% CI: 105.6 to 116.9; 23.1), pacemaker insertion (34.2%, 95% CI: 32.6 to 35.8; 7.5), and defibrillator insertion (94.0%, 95% CI: 88.5 to 100.1; 13.2) (Table 3).

### 3.4. CVD Changes during the COVID-19 Pandemic in View of Pre-Pandemic Trends

Considering the pre-pandemic perennial trends, the significant declines in Y1 observed in STEMI, AF, CHF, CVA, CABG, and defibrillator insertion followed a declining pre-pandemic trend, while the decline observed in non-STEMI contrasted with an increasing trend during 2012–2019. The decline in catheterization, ablation, and pacemaker insertion occurred after a relatively stable trend during the 8 years preceding the pandemic (Figure 1).

When comparing the observed rates in Y1 with the expected rates according to perennial pre-pandemic trends, the rates observed in Y1 for most diagnoses and procedures (STEMI, non-STEMI, AF, catheterization, CABG, pacemaker implantation) were lower than expected based on pre-pandemic trends (*p* < 0.001). For CHF, CVA, and ablation, although decreases from the baseline were observed in Y1, these rates were similar to what was expected from pre-pandemic trends. The observed rate for defibrillator insertion was even higher than what was expected without the ‘disruption’ of the COVID-19 pandemic. 

The increases demonstrated in Y2 of the pandemic also differed in relation to pre-pandemic trends. STEMI, catheterization, and CABG returned to expected rates, non-STEMI and AF were lower than expected, while CHF, CVA, pacemaker insertions, ablation, and defibrillator implantations were more than expected.

## 4. Discussion

In this retrospective, population-based study carried out on ~2.7 million Israeli adults, significant declines were observed in Y1 of the outbreak of the COVID-19 pandemic in all CVD diagnoses and procedures of interest compared with the year prior to the pandemic (2019). In relation to pre-pandemic perennial trends, these rates were within the expected range (CHF, CVA, and ablation), less than was expected (STEMI, non-STEMI, AF, catheterization, CABG, and pacemaker insertion) or more than was expected (in the case of defibrillator insertion). This means that some trends actually remained relatively unchanged (CHF, CVA, and ablation) or increased (defibrillator insertion).

In the second year of the pandemic, an increase was found in all diagnoses and procedures in comparison to Y1, but they differed from pre-pandemic trends. STEMI, catheterization, and CABG returned to expected rates; non-STEMI and AF were lower than expected; and CHF, CVA, pacemaker insertion, ablation, and defibrillator insertion rates were higher than expected. 

The decline in several cardiovascular diagnoses observed in the first year of the pandemic is consistent with those described in many previous reports [9,13,14]. In a survey by the European Society of Cardiology among 3101 cardiologists and cardiovascular nurses across six continents, approximately 80% reported a reduction in STEMI presentations, with a higher rate of delayed presentation during the COVID-19 era [15]. A significant reduction in acute cardiovascular hospitalizations was reported in the USA during the first phase of the COVID-19 pandemic [13]. Based on the Kaiser Permanente system, Solomon et al. reported a decline in hospitalization for STEMI and non-STEMI during the COVID-19 pandemic in a large, diverse population in California [9]. A decline in admissions to coronary care units for STEMI or non-STEMI during the pandemic was also demonstrated in Italy [16], Austria [17], England [14], and Israel [10]. The decrease in diagnoses of AF that was observed in this study was similar to the decrease found in the U.S. by Hernandez et al., 2023 [18] and in Denmark by Holt et al., 2020 [19]. While these studies evaluated the incidence of AF during a narrow timeframe surrounding the outbreak of the COVID-19 pandemic, this study demonstrated a reduction in AF diagnoses during the entire year following the outbreak. The consequence of the pandemic on CVA and CHF diagnoses is not clear-cut. The reduction observed in this study in CVA diagnoses is consistent with that described in most previous publications [20,21,22] and contradicts some others [23,24].

For the procedures, this study found that the reduction in catheterization, CABG, and defibrillator insertion in the first year of the pandemic was consistent with that described in previous reports [25,26]. Furthermore, previous studies also found significant decreases in pacemaker treatments and ablations [25], while these declines were smaller in this study.

Though an actual decline in the incidence of cardiovascular diagnosis and procedures during COVID-19 could not be ruled out, it is widely believed that the sharp reductions were mostly related to avoidance of seeking medical assistance during the pandemic [6,27]. The reduction in procedures during the first year of the pandemic may be explained by the postponement of most elective services [28], especially in procedures such as catheterization, CABG, and pacemaker insertion, where rates dropped lower than the expected rates based on pre-pandemic trends.

Few previous studies have examined the incidence rates of cardiovascular diagnoses and procedures in the year following the COVID-19 pandemic (Y2) separately. For several of the diagnoses and procedures, increases were found between Y2 and Y1 (non-STEMI [29], catheterization [30], CABG, and ablation: Tien et al., 2023 [31]). For the other diagnoses and procedures, little is known about how Y2 of the COVID-19 pandemic affected incidence rates. 

The return to pre-pandemic normalcy for most cardiovascular diagnoses and procedures found in this study during the second year of the pandemic may be related to a return to normal life due to the rapid initiation and extensive rollout of the COVID-19 vaccination program in Israel [32]. 

There could be several possible reasons for the sharp increases observed in ablation, pacemaker insertions, and defibrillator treatments during the second year of the pandemic: (1) An accumulation of procedures (mostly elective) that were postponed during Y1 of the pandemic could have led to the increase observed in those procedures. (2) It is also possible that sub-optimal acute cardiac care given during Y1 of the pandemic (due to deferment or delay in treatment for ischemic patients) led to increased ischemic damage, resulting in increased myocardial dysfunction and risk of arrhythmia, which resulted in the need for defibrillator implants. This may also explain the finding of a greater-than-expected increase in incidences of CHF during Y2. (3) A change in professional guidelines in 2020 led to the expansion of indications for performing ablations in patients with AF, and this may have contributed to the increase observed in Y2 compared to the annual trend. Specifically, the change which was stated in the European Society of Cardiology’s documents [33] recommended performing ablation in patients with symptomatic atrial fibrillation even before drug therapy failure (recommendation class: IIa). This change was based on several large, randomized studies showing the efficacy and safety of ablation, and dramatically affected the incidence of ablations. Future studies are required to evaluate the continuation of these trends in the coming years, to put these increases in Y2 into the context of the overall trends for these procedures.

While most previous studies compared the COVID-19 period to a prior short period close to the outbreak [6], this study described the incidence of cardiovascular diagnoses and procedures as part of a trend throughout a whole decade. The previous studies that compared the COVID-19 period with a long pre-pandemic period have been mostly limited to a single outcome [34] or a small sample size [17,34] or were based on information that was documented during hospitalizations without considering community clinic records [8,16]. Looking at a whole decade, this study enables health professionals to estimate the consequences of the pandemic on cardiovascular outcomes more accurately and, to a large extent, to rule out the influence of secular trends or random changes. For instance, the decline observed in non-STEMI in early 2020, which did not coincide with the trend occurring during the preceding 8 years, can be explained mainly by the outbreak of COVID-19. On the other hand, the decline observed in STEMI, AF, and CABG, as a continuation of the trend during the years 2012–2019, may partially reflect a continuation of pre-pandemic trends. 

A major strength of this study is the availability of an extensive population-based database that includes longitudinal data from both hospitals and community clinics. This provides an up-to-date picture of the trends in the incidence of cardiovascular diagnoses and procedures in Israel over the entire last decade and assesses the impact of the COVID-19 pandemic on cardiovascular diagnoses and procedures as part of a long-standing trend. The main limitation of this study is the possibility of missed diagnoses of cardiovascular diseases among those who refrained from seeking medical assistance during the pandemic. This may result in an underestimation of incidence rates. Additionally, and more generally, the reliance on ICD-9 codes makes it prone to coding bias. However, procedure codes are less likely to be miscoded, and the combination and validation of data from both hospitals and community clinics minimize this risk. Also, this study is limited to the Israeli population. Incidence trends of cardiovascular diagnoses and procedures during 2012–2021 and the impact of the COVID-19 pandemic on incidence rates may differ in other countries. However, observed pre-pandemic trends—a decrease in STEMI and an increase in non-STEMI—were also demonstrated in earlier years (2000–2008) in the USA [35]. This may indicate that these overall trends may not be specific to the Israeli population, but more research needs to be conducted in other counties and during similar time frames in order to understand if these findings could be universal. Finally, the challenges posed by the inherent limitations of retrospective studies may be heightened during a pandemic period, making interpretations more difficult.

## 5. Conclusions

With the outbreak of the COVID-19 pandemic, secular trends in several cardiovascular diagnoses and procedures were interrupted, with different impacts on various outcomes. Possible long-term consequences of the pandemic should be followed and taken into account by health policymakers.

## Figures and Tables

**Table 1:** Study population by year, sex, and age.

Year	N * (Millions)	Women (%)	Mean Age (SD)
2012	2.43	52.1%	49.7 (0.03)
2013	2.48	52.1%	49.7 (0.03)
2014	2.52	52.1%	49.7 (0.03)
2015	2.56	52.0%	49.7 (0.03)
2016	2.59	52.0%	49.7 (0.02)
2017	2.63	52.0%	49.8 (0.02)
2018	2.66	52.0%	49.9 (0.01)
2019	2.69	52.0%	50.0 (0.03)
2020	2.73	51.9%	50.1 (0.02)
2021	2.76	51.9%	50.1 (0.02)

* The calculation is based on the average number of valid members over a 12-month period from March of a specific year to February of the following year.

**Table 2:** The difference in annual incidence rates of cardiovascular diagnoses during the COVID-19 pandemic between the year 2019 and the preceding 8 years (per 100,000 patients).

Year	STEMI	Non-STEMI	AF	CHF	CVA
2012	128.3 (123.8, 132.8)	174.4 (169.2–179.8)	392.4 384.6–400.4	525.0 (516.0, 534.2)	430.1 (421.9, 438.4)
2013	129.5 (125.1, 134.1)	188.2 (182.8–193.7)	379 (371.4, 386.8)	456.1 (447.7, 464.6)	422.6 (414.5, 430.8)
2014	120.7 (116.4, 125.1)	190.7 (185.4, 196.2)	367.6 (360.2, 375.2)	426.9 (418.9, 435.1)	394 (386.2, 401.8)
2015	112 (107.9, 116.2)	182.6 (177.4, 187.9)	377.6 (370.1, −385.2)	409.4 (401.6, 417.3)	375 (367.5, 382.6)
2016	114.9 (110.8, 119.1)	188.8 (183.5–194.2)	381 (373.6, 388.6)	412.9 (405.2, 420.9)	376.6 (369.1, 384.1)
2017	107.4 (103.5, 111.5)	193.6 (188.4, 199.0)	366.5 (359.2, 373.9)	405.3 (397.7, 413.1)	356.9 (349.7, 364.2)
2018	112 (108.0, 116.1)	208.2 (202.7, 213.7)	364.8 (357.6, 372.2)	409.7 (402.0, 417.4)	376.9 (369.6, 384.4)
2019 (baseline)	118.2 (114.1, 122.3)	218.8 (213.3, 224.5)	362.1 (354.9, 369.4)	431.1 (423.3, 439.0)	380.3 (372.9, 387.7)
2020 (Y1)	98.9 (95.2, 102.7)	182.9 (177.9, 188.1)	311.1 (304.5, 317.8)	397.5 (390.0, 405.0)	361.3 (354.2, 368.5)
2021 (Y2)	101.8 (98.1, 105.6)	206 (200.7, 211.4)	334.9 (328.1, 341.8)	447.5 (439.6, 455.4)	388.7 (381.4, 396.1)
Y1 vs. baseline					
n	−19.3 (−24.8, −13.7)	−35.9 (−43.4, −28.4)	−51.0 (−60.7, −41.2)	−33.6 (−44.4, −22.8)	−19.0 (−29.2, −8.7)
%	−16.3% (−16.6, −16.1)	−16.4% (−16.6, −16.2)	−14.1% (−14.2, −14.0)	−7.8% (−7.9, −7.7)	−5.0% (−5.0, −4.9)
Y2 vs. baseline					
n	−16.4 (−22.0, −10.8)	−12.8 (−20.6, 5.1)	−27.2 (−37.1, −17.3)	16.4 (5.3, 27.2)	8.4 (−2.0, 18.8)
%	−13.9% (−14.1, −13.7)	−5.9% (−5.9, −5.8)	−7.5% (−7.6, −7.5)	3.8% (3.8, 3.9)	2.2% (2.2, 2.3)

**Table 3:** The difference in annual incidence rates of cardiovascular procedures during the COVID-19 pandemic between the year 2019 and the preceding 8 years (per 100,000 patients).

Year	Catheterization	CABG	Ablation	Pacemaker	Defibrillator
2012	309.7 (302.7, 316.8)	53.2 (50.3, 56.2)	22.7 (20.8, 24.6)	28.9 (26.8, 31.1)	23.3 (21.4, 25.3)
2013	314.9 (308.0, 322.0)	54.3 (51.5, 57.3)	14.4 (13.0, 16.0)	23.7 (21.9, 25.7)	15.8 (14.3, 17.5)
2014	299.1 (292.4, 306.0)	49.1 (46.4, 51.9)	13.9 (12.5, 15.5)	19.4 (17.8, 21.2)	12.0 (10.7, 13.4)
2015	296.3 (289.7, 303.1)	37.4 (35.0, 39.8)	13.3 (11.9, 14.7)	16.7 (15.2, 18.4)	11.2 (10.0, 12.6)
2016	321.0 (314.2, 328)	38.6 (36.2, 41.1)	14.2 (12.8, 15.7)	21.0 (19.3, 22.8)	10.6 (9.4, 12.0)
2017	325.7 (318.8, 332.7)	37.5 (35.2, 39.9)	12.0 (10.7, 13.4)	20.5 (18.8,22.3)	10.1 (8.9, 11.4)
2018	326.6 (319.8, 333.6)	43.2 (40.8, 45.8)	16.2 (14.8, 17.9)	22.5 (20.8, 24.4)	11.7 (10.5, 13.1)
2019 (baseline)	327.2 (320.4, 334.1)	42.3 (39.9, 44.8)	20.8 (19.1, 22.6)	22.0 (20.3, 23.8)	14.0 (12.6, 15.5)
2020 (Y1)	115.5 (111.5, 119.6)	9.4 (8.3, 10.7)	16.4 (14.9, 18.0)	13.4 (12.0, 14.8)	12.3 (11.0, 13.6)
2021 (Y2)	311.4 (304.9, 318.1)	36.0 (33.8, 38.3)	43.9 (41.5, 46.5)	29.5 (27.5, 31.6)	27.2 (25.3, 29.2)
Y1 vs. baseline					
n	−211.7 (−219.7, −203.7)	−32.9 (−35.6, −30.2)	−4.4 (−6.7, −2.1)	−8.6 (−10.4, −6.4)	−1.7 (−3.7, 0.3)
%	−64.7% (−65.2, −64.2)	−77.7% (−79.2, −76.2)	−21.2% (−22.0, −20.4)	−39.3% (−40.7, −37.9)	−12.5% (−13.1, −12.0)
Y2 vs. baseline					
n	−15.8 (−25.3, −6.3)	−6.3 (−9.6, −3.0)	23.1 (20.1, 26.2)	7.5 (4.8, 10.2)	13.2 (10.8, 15.6)
%	−4.8% (−4.9, −4.8)	−15.0% (−15.4, −14.6)	111.1% (105.6, 116.9)	34.2% (32.6, 35.8)	94.0% (88.5, 100.1)

## Data Availability

The data are not publicly available due to privacy restrictions.

## Supplementary Materials

The following supporting information can be downloaded at: https://www.mdpi.com/article/10.3390/jcm13020476/s1, Table S1: ICD-9 codes for cardiovascular diagnoses and procedures.
