# Supplementary material for: Trends in the Incidence of Cardiovascular Diagnoses and Procedures over the Years 2012–2021 in Israel: The Impact of the COVID-19 Pandemic
Source: J Clin Med. 2024 Jan 15;13(2):476. doi: 10.3390/jcm13020476 (PMC10816154; doi:10.3390/jcm13020476)
Supplement: Supplementary file 1 [file jcm-13-00476-s001.zip › jcm-2794187-supplementary.pdf]

Supplementary Table S1: ICD-9 codes of Cardiovascular diagnosis and treatments.

**Diagnosis**	**ICD-9 codes**	**Source**
ST-elevation myocardial infarction (STEMI)	410.1%-410.6%, 410.8%	Hospital EMRs
Non-ST-elevation myocardial infarction (non-STEMI)	410.7%, 410.9%<br>410	Hospital EMRs
Atrial fibrillation (AF)	427.3, 427.31, 427.32, K78	Community and Hospital EMRs
Congestive heart failure (CHF)	428.0, 428.1<br>428.2, 428.20, 428.21, 428.22, 428.23<br>428.3, 428.31, 428.32, 428.33<br>428.4, 428.40, 428.41, 428.42, 428.43,<br>112.1, 112.3, 112.9<br>398.91, 402.01, 402.11, 402.91, 404.01, 404.03, 404.11, 404.13, 404.91, 404.93, 416. 9, 514	Community and Hospital EMRs
Cerebrovascular accident (CVA)	430, 431, 432, 433%, 434.91, 436, 438.0, 438.1%, 438.2%	Community and Hospital EMRs
Ablation	37.34	Hospital EMRs
Catheterization	0066, 0040, 0041, 0042, 0043, 0044, 0045, 0046, 0047, 0048	Hospital EMRs
Coronary artery bypass graft (CABG)	36.1, 36.10, 36.15, 36.16, 36.11, 36.12, 36.13	Hospital EMRs
Pacemaker	0050, 0053, 37.71, 37.73, 37.81, 37.82, 37.83	Hospital EMRs
Defibrillator	0051, 0054, 37.94, 37.98	Hospital EMRs
